# Strategies to Improve Patient Flow in the Emergency Department during the COVID-19 Pandemic: A Narrative Review of Our Experience

**DOI:** 10.1155/2022/2715647

**Published:** 2022-10-07

**Authors:** Ali S. Al-Shareef, Azzah Al Jabarti, Kholoud A. Babkair, Maan Jamajom, Abduallah Bakhsh, Syed Sameer Aga

**Affiliations:** ^1^Department of Emergency Medicine, King Abdulaziz Medical City, Ministry of National Guard-Health Affairs, Jeddah 21423, Saudi Arabia; ^2^King Abdullah International Medical Research Centre, King Abdulaziz Medical City, Ministry of National Guard-Health Affairs, Jeddah 21423, Saudi Arabia; ^3^College of Medicine, King Saud Bin Abdulaziz University for Health Sciences, King Abdulaziz Medical City, Ministry of National Guard-Health Affairs, Jeddah 21423, Saudi Arabia; ^4^Department of Emergency Medicine, King Abdulaziz University Hospital, Jeddah, Saudi Arabia

## Abstract

**Objective:**

The COVID-19 pandemic has resulted in a tremendous strain on the global healthcare system. Emergency departments worldwide have been challenged to the extreme end. This has led clinicians and policy creators to rearrange patient flow pathways for an efficient emergency department (ED).

**Methods:**

It was reported according to our experience of utilizing a novel strategy to enhance patient flow while reducing the risk of infection transmission among patients and healthcare staff. This included the development of three layers of triage. First, an outer checkpoint prior to the hospital entrance was established to identify eligible patients for emergency department visits. The second layer of triage is located at the ED entrance to direct patients either to the respiratory or nonrespiratory care area to identify potentially infected patients and the third is the routine full triage activity. Then, after completing a clinical assessment in the ED, physicians determine the need for an inpatient isolation unit, a nonisolation inpatient unit, or discharge. Moreover, examples of additional measures were substantial changes to shift schedules; rerouting ambulance crews with COVID patients to inpatient beds directly bypassing the ED; controlled use of personal protective equipment (PPE); and implementing appropriate COVID-19 screening tests.

**Results:**

During the peak of the pandemic, our strategies achieved favorable results and minimized unnecessary ED visits without any patient complications.

**Conclusion:**

This current study provides a set of newly developed steps and procedures that can be further control patient flow pathways and maintain a low risk of infection transmission to a manageable level for an efficient ED.

## 1. Introduction

The rapid spread of severe acute respiratory syndrome coronavirus-19 (SARS-CoV-2) demands urgent guidance for clinicians and policymakers faced with this novel illness, particularly healthcare workers in emergency departments (EDs). Emergency Department health care workers are the frontline of hospital-and-community-based care, serving as the main points of contact for patients [1].

In early March 2020, Saudi Arabia had the first laboratory-confirmed COVID-19 case, followed by multiple positive cases. Most of them had links to international travel [2]. The government of Saudi Arabia's had escalated its risk assessment and public health response by implementing a multipronged surveillance and containment strategy [3].

The National Guard Health Affairs, King Abdulaziz Medical City in the western region, is a 751-bed tertiary medical center in Saudi Arabia [4]. The hospital's Emergency Department serves approximately 70,000 adult and pediatric patients annually. The evolving outbreak situation and case definitions (Appendix 1) presented challenges for implementing logistics and workflow while maintaining continuity of care. Our experience in combating COVID-19 may have positive implications for emergency departments in preparing for and responding to this or a similar future pandemic.

This article presents our implemented strategies for managing patient flow while reducing the risk of infection transmission among patients and healthcare staff.

## 2. Triage

**Figure 1:**
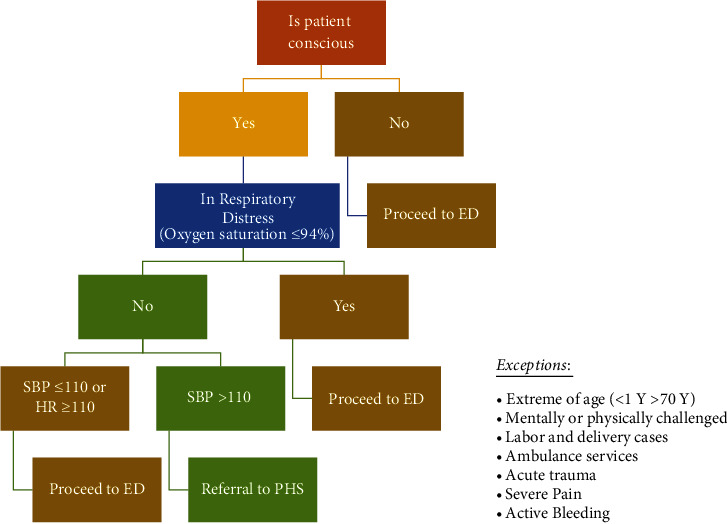
Triage process flow at the main checkpoint.

**Figure 2:**
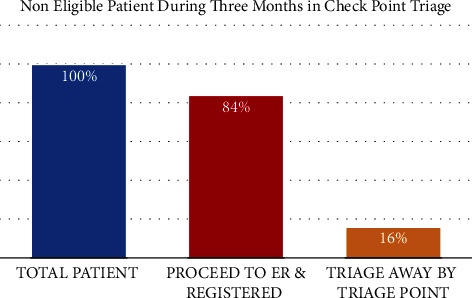
Triage away patients during three months period at checkpoint triage.

Triage aims to stratify patients' presentations and prioritize them accordingly as a way of allocating limited resources, such as staff and physical space, based on their clinical needs [5]. In our setting, before the pandemic, we only had one main ED triage. To mitigate the situation at the beginning of the outbreak, we decided to implement multiple layers of triage as follows:

- Forward Triage (Known as Check Point): the decision to close one of the two main car gates was made by the hospital administrators. The ED allocated its first forward triage (Check Point) at the entrance of the main gate. The checkpoint has a special lane (emergency lane) marked on the road for easy visual identification by incoming patients. Triage in this area is a simple and rapid process carried out by trained paramedics and supported by senior ED physicians via phone directives. Based on the clinical algorithm, patients will be evaluated while they are in their cars (Figure 1). During the peak of the pandemic, triage in this area was able to achieve favorable results and minimize unnecessary ED visits without any significant patient complications (Figure 2). Patients with low acuity were directed to our primary healthcare center which is close to our ED.
- Booth Screening and triage: this is a walk-in second-layer triage located at the main entrance of the ED, operating around the clock. The main goals were to measure patients' temperature, provide medical face masks, and assess the risk of Middle East respiratory syndrome (MERS)/COVID-19 patients based on the acute respiratory infection risk (ARI) score (Appendix 2). The patients later are divided into two streams of flow; low risk and high risk (discussed in the patient streaming section).
- Main ED Triage: this is our main daily routine triage process wherein the Canadian Triage Acuity Scale (CTAS) was utilized (Table 1) and the ARI scale was remeasured for the second time at the main physical triage room for confirmation.

## 3. Patient Streaming

**Figure 3:**
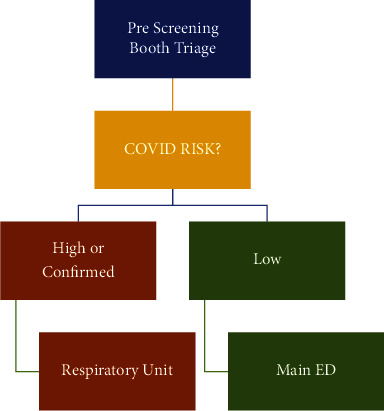
Prescreening visual triage booth.

Streaming is the process of allocating similar patients' disease severity or nature of the complaint to a particular work stream [6]. In this particular pandemic, it was aimed to segregate patients from booth screening to high COVID-19 risk versus low risk (Figure 3). Upon walk-in to booth triage, patients were directed to either the respiratory unit or the main ED:

- Respiratory Unit: this is a newly created unit adjacent to the main ED building aimed to serve high-risk or confirmed COVID-19 cases. The unit was operated by the emergency department twenty-four hours/seven days a week. The unit has the capacity of seven negative pressure isolation rooms with controlled entrances and exits. The main purpose of this unit is to manage confirmed or high-risk suspected patients. The unit is served with an on-site pharmacy for discharge medications as well as patient services to serve discharged patients with a medical report, and sick leave to minimize exposure risk inside the hospital.
- Main ED: this is a 35-bed-capacity with two negative pressure rooms capable of managing critically ill patients and equipped with 15 HEPA Air purifier machines distributed within the department. The critically ill suspected cases such as CTAS I &II were placed in one of these negative pressure rooms for critical care management till their medical clearance as per infection control protocol or board-certified emergency physician decision.

## 4. Manpower (Physicians and Residents)

Frontline healthcare workers are at high risk of infection, contributing to the further spread of infection [7]. Initial estimates suggest that frontline healthcare workers could account for 10–20% of all COVID-19 diagnoses [8, 9].

Our ED physician's manpower was split, with senior and junior doctors rostered into four modular teams working in 8-hour shifts to prevent cross-exposure. Until writing this article, no definitive hospital-acquired infection among our ED physicians was detected.

## 5. ED Consultation and Admission

Emergency Department (ED) overcrowding is a worldwide problem that poses a threat to patient safety by causing treatment delays and increasing mortality [10, 11]. A prolonged ED length of stay (LOS) contributes to overcrowding, and its consequences include decreased quality of treatment, misuse of medical resources in the ED, decreased patient satisfaction [12], and an increased risk of infection transmission. It has been studied once that admission decisions regarding medical patients made by emergency physicians without specialty consultations in the ED reduce the LOS without a significant negative effect on mortality or hospital LOS [13].

Our hospital designated a special inpatient COVID-19 ward for suspected and another one for confirmed patients as well as designated medical teams.

**Figure 4:**
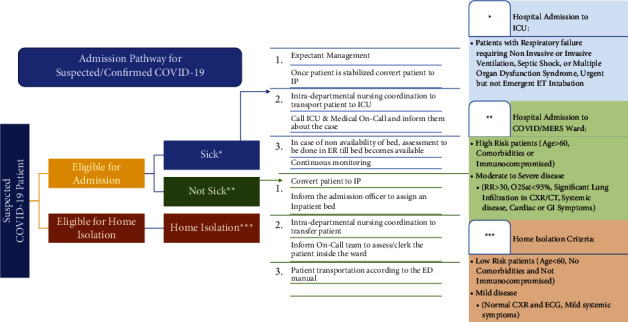
ED admission pathway of suspected/confirmed COVID-19 cases.

The ED coordinated a clinical admission pathway (Figure 4). This pathway has privileged ED consultants to admit suspected or confirmed cases to the designated wards without the physical presence of consulting services in the ED.

**Figure 5:**
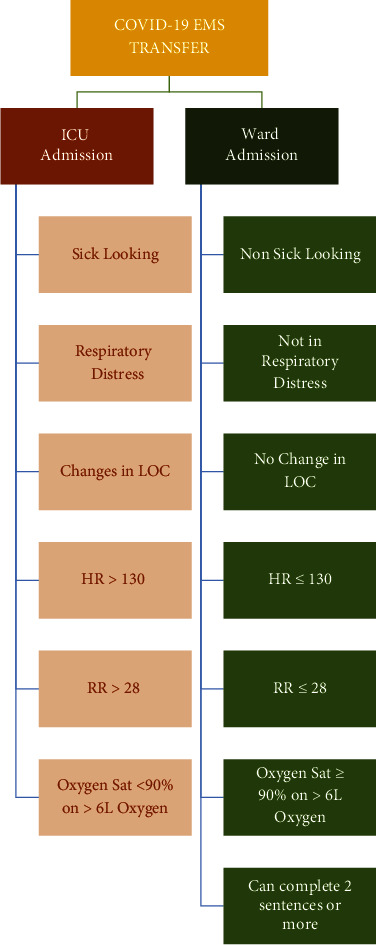
COVID-19 EMS transport algorithm.

Moreover, ED management provided a coordination point and safe transportation to the hospital for those confirmed home isolated PCR positive cases in need of hospital admission. These patients have been identified by the daily Infection Prevention Control Department physicians' (IPC) telemedicine assessment. Transportation was overseen by a multidisciplinary team, also known as the command center team, who coordinated the dispatch of our EMS providers to escort patients by well-trained and equipped ambulance service teams directly to the designated wards through a well-coordinated process without passing through ED. The patients' transportation and assessment follow the online emergency physician's medical direction and a clinical pathway (Figure 5). There were a few occasions, where hospital critical care beds were full, and patients needed to be resuscitated and escorted to the ED rather than COVID wards or the Intensive care unit (ICU).

## 6. COVID-19 Testing

During the initial outbreak, COVID-19 nasopharyngeal swabs were only performed in the ED, which posed an increased length of stay and potential risk of infection transmission. However, early on, the institute adopted two strategies that showed a positive impact on the patient flow process.

**Figure 6:**
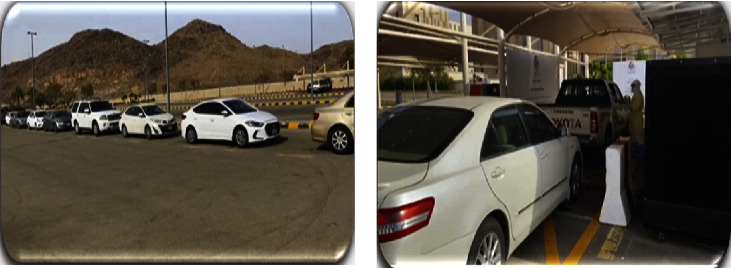
Drive-through Center of the ED department in NGHA.

**Figure 7:**
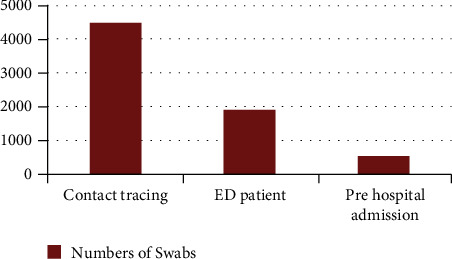
COVID-19 drive-through cases distributed from May to December 2020.

- Drive-through COVID-19 testing (Figure 6): testing for COVID-19 while patients were in their cars, not only decreased the risk of infection transmission but also diverted unnecessary ED visits (Figures 6 and 7). The drive-through station is located close to the ED building. After applying the testing criteria (Table 2), the process of booking and patient organization was done through department coordinators.
- Rapid PCR COVID-19 test (GeneXpert): a few months after the outbreak, the institute laboratory department installed a rapid PCR COVID- 19 test with a turnaround time of less than 2HR compared to our previous PCR test (Abbott and Altona) which takes an average turnaround time of 18HR [14].

## 7. Personal Protective Equipment (PPE) Supply

A Shortage of PPE is reported worldwide due to a rise in demand, panic buying, and irrational use (WHO, 2020 g) [15]. The global shortage of PPE was experienced during the Ebola outbreak of 2014–2016 in West Africa, which resulted in a high number of infected HealthCare Workers HCWs (900 infected, 500 deaths) [16]. Our ED provides PPEs not only to the permanent staff who work in the ED but also to those who respond to ED calls from other specialties. Therefore, the following strategies were implemented to optimize the supply of PPE during potential shortages:

### 7.1. Coordination with the Hospital Logistic Department

The hospital COVID-19 crisis committee (ED represented) was meeting on a daily basis (virtually) to discuss the crisis, which included the supply-demand situation. The majority of challenges were solved during this meeting. Moreover, ED did have direct access to the logistic department around the clock in case of real shortages.

### 7.2. Optimizing the Supply of PPE

ED leadership has worked closely with the Infection Prevention Control Department and hospital logistics to continuously evaluate the demand and supply of PPE while adapting the CDC strategies for optimizing supplies.

Reference [17]. These strategies offer a continuum of options using the framework of surge capacity when PPE supplies are stressed, running low, or absent. Furthermore, a log sheet was created and placed in the ED storeroom to track the use of PPE. For further clarification, below are the descriptions of each capacity:

#### 7.2.1. Conventional Capacity

Strategies that are in place as part of general infection prevention and control plans. This includes using physical barriers, limiting the number of unnecessary hospital visits, using telemedicine whenever possible, limiting all healthcare providers not directly involved in patient care, and limiting visitors to the facility.

#### 7.2.2. Contingency Capacity

Strategies used during the period of anticipated PPE shortage. This includes selectively canceling elective and nonurgent procedures and appointments for which PPE is typically used by healthcare practitioners (HCP).

#### 7.2.3. Crisis Capacity

Strategies are used when supplies cannot meet the department's current PPE utilization rate. This includes canceling all elective and nonurgent procedures and appointments for which PPE is typically used by HCP.

## 8. ED Staff Psychological Safety during COVID-19 Pandemic

The Consequences of COVID-19 on mental health have been observed and reported worldwide. In the United States, Panchal et al. [18] found that 4 out of 10 people were reporting increased symptoms of depression and anxiety, with a negative impact on mental health and well-being. One share that was relatively stable in 2019 was that 1 in 10 people was reporting such symptoms. Healthcare workers, especially the front-liners in the Emergency Department suffered the fear of transmitting the disease to their loved ones and had to deal with self-isolation. All of that and more mandated several steps to be taken as follows:

- Initiation of an Emergency Department support group, an idea that was led by the Emergency Residency Program and empowered by the ED Chairman and the program director. The main function of this group is to provide informal moral, psychological treatment when needed, and logistical PPE supplies support to any healthcare worker who contracts COVID-19 or who needs isolation because of contact with confirmed COVID-19 cases.
- Early identification of the psychological effects of COVID-19 by regular check-ups on staff allows them to provide the appropriate support when needed.
- By providing more leniency toward giving more off times to those who start to experience symptoms of burnout.
- Providing a chance for staff to visit their families every few weeks by offering COVID-19 testing and arranging their off times internally.

The aforementioned measures were merely unstructured plans in a trial to provide humanitarian support to the health care workers who were the main force against fighting COVID-19.

## 9. Future Directions

Based on the detailed experience mentioned above, the following areas of improvement were identified:

- Many employees were unfamiliar with the use of certain PPE devices, such as the powered air-purifying respirator (PAPR), and others with the fitting standard N95 masks. Therefore, the need for periodic PPE training courses was recognized.
- There is a need to revisit our disaster preparedness plan and build integrated strategic, tactile, and operational plans for pandemic preparedness.
- PPE demand and supply chains need to be reviewed and future demands need to be strategically assured.
- ED policy regarding communication with patients and families at home and or inside ED assessment rooms should be updated using the advancement in health telecommunication supported by the hospital's higher administration in terms of resources, legal responsibility, and proper documentation.
- A significant proportion of frontline medical workers experienced psychological symptoms during the COVID-19 outbreak. Therefore, proper disaster preparedness, including training, stress management courses, and creating solid support groups is needed.
- Better structure of forwarding triage for different types of disasters. Our forward triage requires support in terms of more manpower and better design to accomplish the required mission.

## 10. Conclusion

It was a great learning opportunity from the experience to review our organizational management with a multidisciplinary team plan. Despite the clear effectiveness of ED strategies to enhance patient flow while reducing the risk of infection transmission among patients and healthcare staff, more improvement and future directions are listed to be highly considered.

## Figures and Tables

**Table 1:** Canadian Triage and Acuity Scale levels, associated times to assessment and treatment, and frequency of assessment.

Level	Cause	Time to physician assessment and treatment	Frequency
I	Resuscitative	Immediately	Continuous care
II	Emergent	Within 15 minutes	Every 15 minutes
III	Urgent	Within 30 minutes	Every 30 minutes
IV	Less urgent	Within 60 minutes	Every 60 minutes
V	Nonurgent	Within 120 minutes	Every 120 minutes
Canadian Association of Emergency Physicians (1998)			

**Table 2:** Criteria for COVID-19 testing at drive-through center.

S. No.	Criteria
1	Patients require elective hospital admission
2	Patient has direct contact with confirmed cases
3	CTAS IV&V who presented to ED with a high acute respiratory infection (ARI), with a score > 4
4	Cases discharged from ED with a physician's decision to swab them

## Data Availability

Raw data are available from the corresponding author upon request.

## Supplementary Materials

Supplementary Materials Appendix 1: Saudi Ministry of Health COVID-19 case Definition. Appendix 2: acute respiratory infection risk score (ARI)-visual triage checklist.

## References

[1] Whiteside T., Kane E., Aljohani B., Alsamman M., Pourmand A. (2020). Redesigning emergency department operations amidst a viral pandemic. *The American Journal of Emergency Medicine*.

[2] Alyami M. H., Alyami H. S., Warraich A. (2020). Middle east respiratory syndrome (Mers) and novel coronavirus disease-2019 (COVID-19): from causes to preventions in Saudi Arabia. *Saudi Pharmaceutical Journal*.

[3] (2021). Official updates coronavirus-COVID-19 in Saudi Arabia. https://covid19awareness.sa/en/home-page.

[4] Ministry of National Guard Health Affairs (Mngha) (2021). Jeddah campus. https://ngha.med.sa/english/MedicalCities/Jeddah/.

[5] Jarvis P. R. E. (2016). Improving emergency department patient flow. *Clinical and Experimental Emergency Medicine*.

[6] Oredsson S., Jonsson H., Rognes J. (2011). A systematic review of triage-related interventions to improve patient flow in emergency departments. *Scandinavian Journal of Trauma, Resuscitation and Emergency Medicine*.

[7] Black J. R. M., Bailey C., Przewrocka J., Dijkstra K. K., Swanton C. (2020). COVID-19: the case for health-care worker screening to prevent hospital transmission. *The Lancet*.

[8] Burrer S. L., de Perio M. A., Hughes M. M. (2020). Characteristics of health care personnel with COVID-19: United States, February 12-April 9, 2020. *MMWR Morbidity and Mortality Weekly Report*.

[9] Lazzerini M., Putoto G. (2020). COVID-19 in Italy: momentous decisions and many uncertainties. *Lancet Global Health*.

[10] Harper A., Jones P., Wimsett J. (2016). The effect of the shorter stays in emergency departments health target on the quality of ED discharge summaries. *Emergency Medicine Journal*.

[11] van der Veen D., Remeijer C., Fogteloo A. J., Heringhaus C., de Groot B. (2018). Independent determinants of prolonged emergency department length of stay in a tertiary care centre: a prospective cohort study. *Scandinavian Journal of Trauma, Resuscitation and Emergency Medicine*.

[12] McCarthy M. L., Zeger S. L., Ding R. (2009). Crowding delays treatment and lengthens emergency department length of stay, even among high-acuity patients. *Annals of Emergency Medicine*.

[13] Choi Y., Jeong J., Kim B.-G. (2020). Admission decisions made by emergency physicians can reduce the emergency department length of stay for medical patients. *Emergency Medicine International*.

[14] Dhibar D., Singh H. (2020). Old test for the diagnosis of novel corona virus infection: role of GeneXpert in COVID-19 testing. *Journal of Family Medicine and Primary Care*.

[15] WHO (2021). Shortage of personal protective equipment endangering health workers worldwide. https://www.who.int/news/item/03-03-2020-shortage-of-personal-protective-equipment-endangering-health-workers-worldwide.

[16] Sharma N., Hasan Z., Velayudhan A., Mangal D. K., Gupta S. D. (2020). Personal protective equipment: challenges and strategies to combat COVID-19 in India: a narrative review. *Journal of Health Management*.

[17] CDC (2021). Optimizing the supply of PPE in Healthcare facilities. https://www.cdc.gov/coronavirus/2019-ncov/hcp/ppe-strategy/strategies-optimize-ppe-shortages.html.

[18] Panchal U., Salazar de Pablo G., Franco M. (2021). The impact of COVID-19 lockdown on child and adolescent mental health: systematic review. *European Child & Adolescent Psychiatry*.

