# Supplementary material for: Strategies to Improve Patient Flow in the Emergency Department during the COVID-19 Pandemic: A Narrative Review of Our Experience
Source: Emerg Med Int. 2022 Oct 7;2022:2715647. doi: 10.1155/2022/2715647 (PMC11410429; doi:10.1155/2022/2715647)

SUPPLEMENTARY MATERIAL

Appendix (1): SAUDI MINISTRY OF HEALTH COVID-19 CASE DEFINITION

(Available at URL: https://covid19.cdc.gov.sa/wp-content/uploads/2020/10/EN_COVID_19_Coronavirus_Disease_Guidelines_v2.0.pdf)


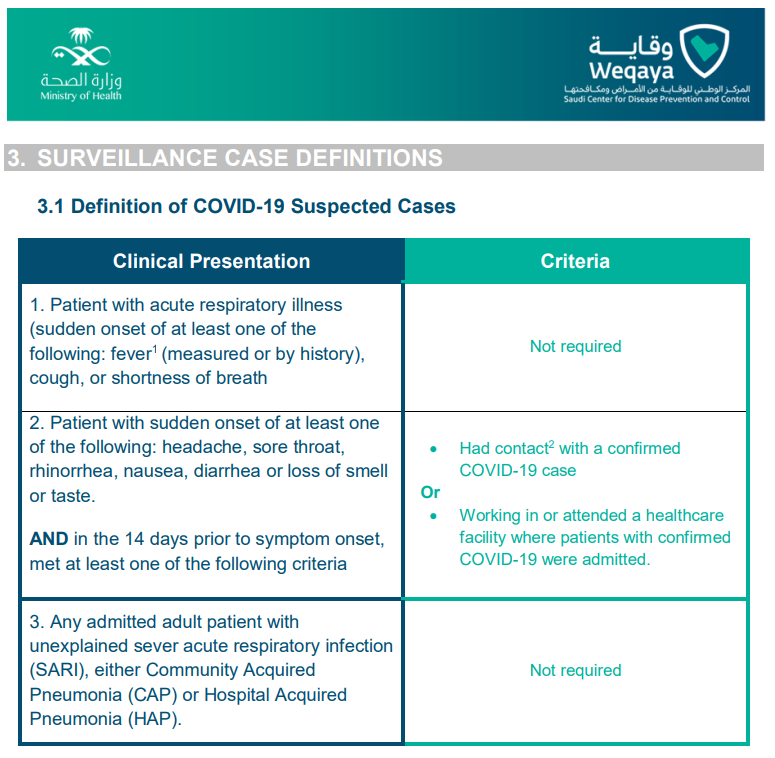


Appendix (2): ACUTE RESPIRATORY INFECTION RISK SCORE (ARI)

(Available at URL: https://www.moh.gov.sa/CCC/healthp/regulations/Documents/Coronavirus%20Disease%202019%20Guidelines%20v1.1..pdf)


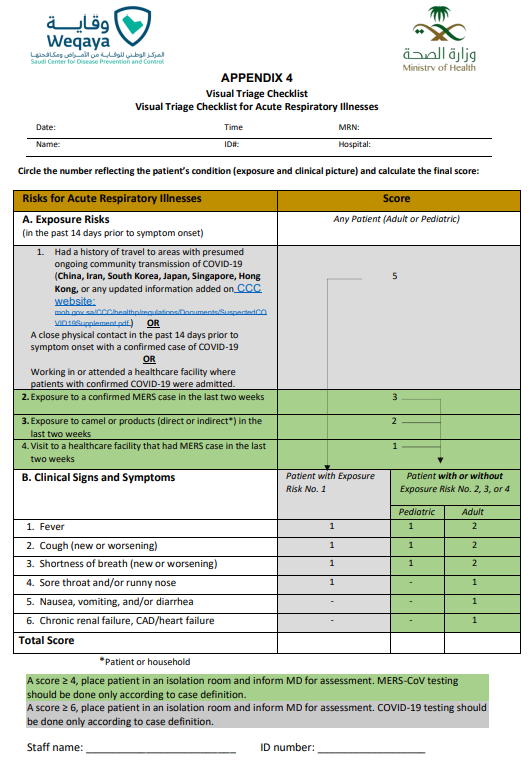

Supplement: Supplementary Materials — Appendix 1: Saudi Ministry of Health COVID-19 case Definition. Appendix 2: acute respiratory infection risk score (ARI)-visual triage checklist. [file 2715647.f1.docx]
